# Construction of immune-related gene pairs signature to predict the overall survival of osteosarcoma patients

**DOI:** 10.18632/aging.104017

**Published:** 2020-11-16

**Authors:** Long-Qing Li, Liang-Hao Zhang, Yan Zhang, Xin-Chang Lu, Yi Zhang, Yong-Kui Liu, Manhas Abdul Khader, Jia-Wen, Tao-Liu, Jia-Zhen Li

**Affiliations:** 1 Department of Orthopaedic Surgery, The First Affiliated Hospital of Zhengzhou University, Zhengzhou, Henan, PR China; 2 Department of Urology, The First Affiliated Hospital of Zhengzhou University, Zhengzhou, Henan, PR China

**Keywords:** immune related gene, tumor immunology, osteosarcoma, TCGA, prognosis

## Abstract

The purpose of this study is to establish the prognosis of osteosarcoma patients based on the characteristics of immune-related gene pairs. We used the lasso Cox regression model to construct and verify the signature consisting of 14 immune-related gene pairs. This signature can accurately predict the overall survival of osteosarcoma patients and is an independent prognostic factor for osteosarcoma patients. For this we constructed a signature-based nomogram. The results of the nomogram show that our signature can bring clinical net benefits. We then assessed the abundance of infiltrating immune cells in each sample, and combine the results of the gene set enrichment analysis of a single sample to explore the differences in the immune microenvironment between IRPG signature groups. The result of gene set enrichment analysis shows the strong relationship between signature and immune system. Finally, we evaluated the relationship between signature and immunotherapy efficiency using algorithms such as TIMI and SubMap to explore patients who might benefit from immunotherapy. In conclusion, our signature can predict the overall survival rate of osteosarcoma patients and provide potential guidance for exploring patients who may benefit from immunotherapy.

## INTRODUCTION

Osteosarcoma is a malignant bone tumor, which is mainly associated with children and adolescents [1]. Today, newly diagnosed osteosarcoma patients are mainly treated by chemotherapy and surgery, and the five-year survival rate can reach 60-70%. However, some patients who are not sensitive to chemotherapy or have metastases only have a five-year survival rate of 20-30%, and new personalized treatment plans need to be developed to improve the prognosis of these patients [2, 3]. Today, the most important guidelines for risk stratification and clinical decision making for patients with osteosarcoma are still clinical characteristics such as metastasis. However, patients with the same clinical characteristics and the same treatment showed very different clinical results [4]. Therefore, we have a need to consider the new prognostic factors to more accurately stratify patients to develop personalized treatment plans.

Today, the immune system is considered to play a vital role in the initiation and development of tumors [5]. Several immunotherapies including immune checkpoint inhibitors have been developed for this characteristic of tumors. Among them, immune checkpoint inhibitors targeting cytotoxic T lymphocyte associated antigen 4 (CTLA-4) and programmed cell death 1 (PD-1) have shown great potential in the treatment of various tumors [6–8]. Related research is also in full swing in patients with osteosarcoma, promising to improve the survival of patients with osteosarcoma [9, 10]. However, new treatment methods require us to have a new understanding of tumors. Unfortunately, the molecular mechanism of tumor immunity in osteosarcoma remains undetermined. In addition, it is important to more accurately identify patients who are more likely to benefit from immunotherapy [11].

With the development of high-throughput gene detection technology and the establishment of large-scale gene expression data sets, researchers can more accurately identify key molecular features and combine them with clinical features to more precisely stratify patients to develop individualized treatment plan [12–14]. Previous research based on gene expression characteristics to develop multiple multi-gene signatures can identify high-risk patients. However, there are differences in the measured gene expression levels due to the different platforms for detecting gene expression. This brings certain difficulties to the comprehensive use of these data [15]. Recently, researchers have provided a new way to solve this difficulty, which is to normalize and scale based on the relative ranking of gene expression levels. This approach has produced reliable results in various studies [16–18]. Therefore, the purpose of this study is to research the value of immune-related gene pairs (IRGPs) in osteosarcoma, in predicting patient-survival and to explore their potential in predicting the effectiveness of immunotherapy.

## RESULTS

### Construction and evaluation of IRGPs signature

**Figure 1:**
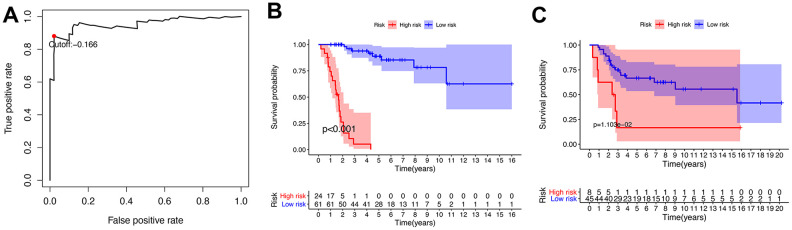
**Establishment and verification of IRGP signature.** (**A**) Time-dependent ROC curve for IRGP signature in the TCGA cohort. The optimal cut-off value of IRGP signature is -0.166, and patients are divided into high-risk group and low-risk group according to the cut-off value (**B**) Kaplan–Meier curves of overall survival according to IRGP signature groups in the TCGA cohort. (**C**) Kaplan–Meier curves of overall survival according to IRGP signature groups in the GSE21257 cohort.

**Figure 2:**
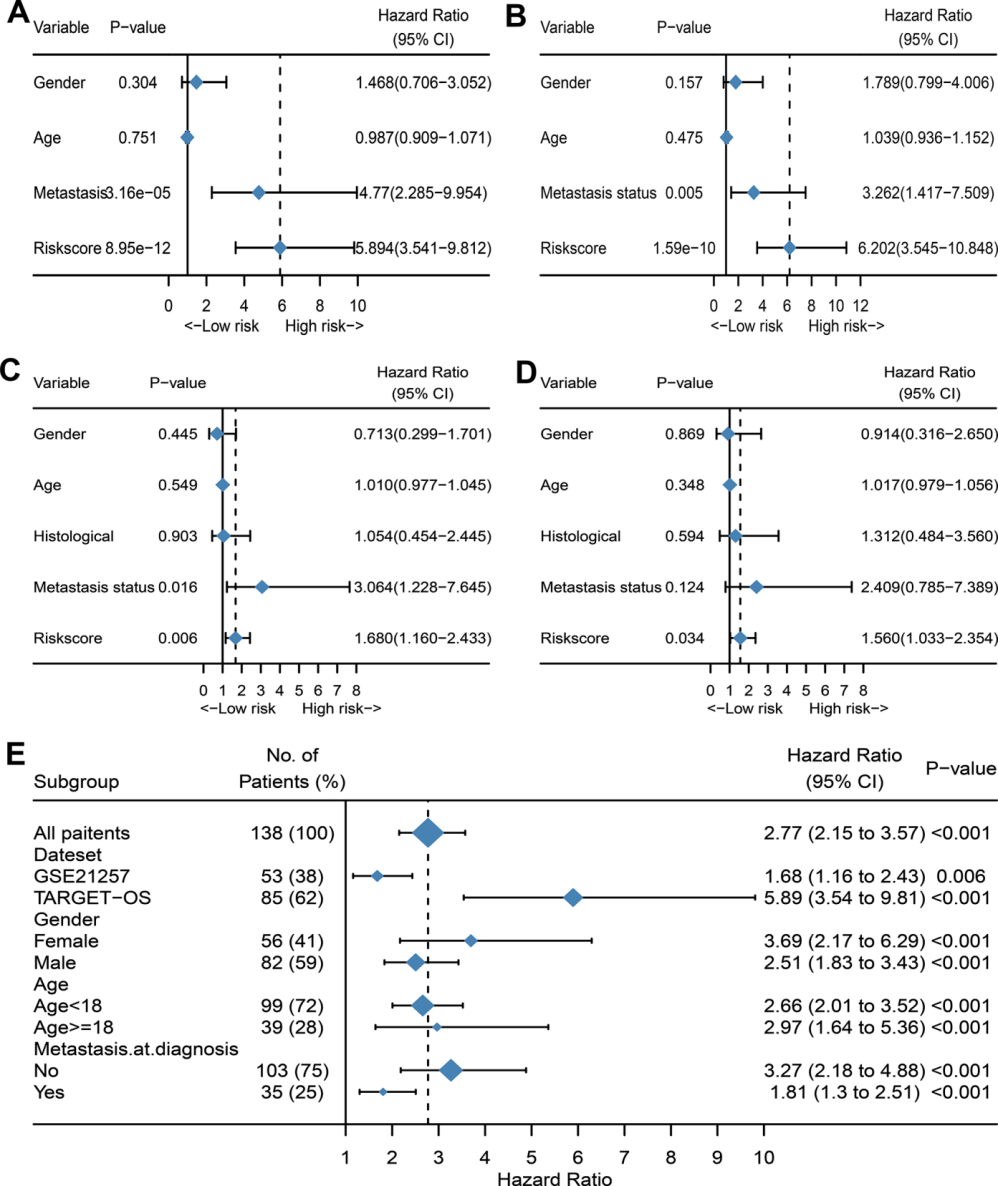
**Evaluate whether IRGP signature is an independent prognostic factor.** (**A**) Forest plot of univariate Cox regression results of IRPG signature and clinical characteristics of TCGA cohort. (**B**) Forest plot of univariate Cox regression results of IRPG signature and clinical characteristics of GSE21257 cohort. (**C**) Forest plot of multivariate Cox regression results of IRPG signature and clinical characteristics of TCGA cohort. (**D**) Forest plot of multivariate Cox regression results of IRPG signature and clinical characteristics of GSE21257 cohort. (**E**) Forest plots of the associations between IRGP and overall survival in various subgroups.

**Figure 3:**
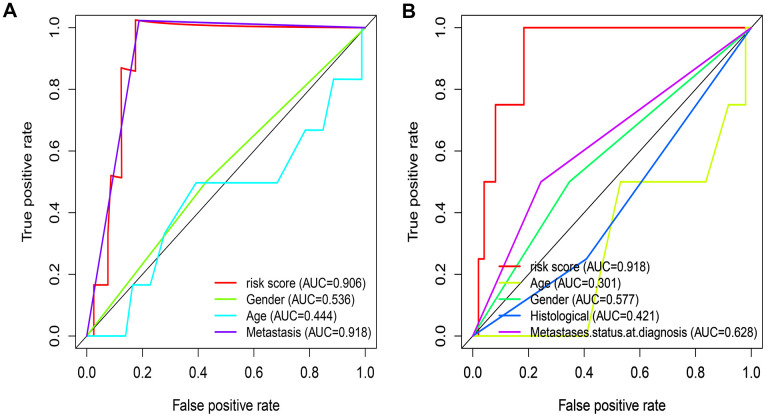
**Evaluate the predictive ability of IRGP signature.** (**A**) ROC curve of clinical characteristics and IRGP signature in TCGA cohort. (**B**) ROC curve of clinical characteristics and IRGP signature in GSE21257 cohort.

A total of 141 patients with osteosarcoma were included in our study. The TCGA cohort has 88 patients and the GSE21257 cohort has 53 patients. Three patients in the TCGA cohort lacked valid clinical information and were therefore not included in survival-related analysis. Table 1 shows the basic characteristics of these patients. A total of 210 immune-related genes are considered to have high variability and used to construct immune-related gene pairs (Supplementary Table 1). The IRGPs with low variation was deleted and a total of 2260 IRGPs were retained for further analysis (Supplementary Tables 2, 3). We selected the TCGA cohort as the training set and identified a total of 32 immune-related gene pairs related to prognosis (P <0.005) (Supplementary Table 4). We used Lasso Cox proportional hazard regression on the training set to define the IRGP signature, and selected the final model consisting of 14 IRGPs. The IRPG signature consists of 21 IRGs as shown in Table 2. We used the time-dependent ROC curve analysis to determine the optimal cut-off value of the IRGP signature. As shown in Figure 1, the optimal cut-off value of the IRGP signature is -1.66. We divided patients into high-risk group and low-risk group according to cut-off value. As shown in Figure 1, compared to patients in the low-risk group the overall survival of patients in the high-risk group is significantly reduced. Subsequently, we conducted univariate and multivariate Cox regression analysis to explore whether IRGP signatures can be used as independent predictors of prognosis. As shown in Figure 2, after adjusting for variables such as metastasis status, the result showed that the IRGP signature is an independent prognostic factor for the TCGA cohort (HR:6.202, 95%CI: 3.545-10.848, P< 0.001). Figure 3 is the receiver working curve of IRGP signature, metastasis status, age and gender. As shown in Figure 3, the area under the curve (AUC) of the IRGP signature is 0.906, which indicates that our signature has excellent predictive ability.

**Table 1:** Summary of clinical characteristics of Osteosarcoma patient data sets in the study.

**Characteristic**	**Training cohort (TCGA n=88)**	**Validation cohort (GSE21257 n=53)**
**Vital status, n (%)**		
Alive	57(64.8)	30(62.3)
Dead	29(33.0)	23(37.7)
Unknow	2(0.2)	NA
**Age, n (%)**		
> = 18	19(21.6)	20(37.7)
<18	69(78.4)	33(62.3)
**Gender, n (%)**		
Male	51(58.0)	34(64.2)
Female	37(42.0)	19(35.8)
**Metastasis, n (%)**		
M0	66(75)	19(35.8)
M1	22(25)	34(64.2)
**Histological, n (%)**		
Osteoblastic	NA	32(60.4)
Other	NA	21(39.6)

NA represents information not available.

**Table 2:** Information on 14 IRGPs.

**Gene pair**	**Gene1**	**Gene2**	**Coefficient**
HLA-DQB1|STC2	HLA-DQB1	STC2	-0.2334234
APOD|CCL2	APOD	CCL2	0.38929255
F2R|SEMA3B	F2R	SEMA3B	-0.0684453
F2R|ANGPTL4	F2R	ANGPTL4	-0.1359965
HCK|PLXNB1	HCK	PLXNB1	-0.5042191
HCK|STC1	HCK	STC1	-0.8295186
RAC3|FGFRL1	RAC3	FGFRL1	0.84832068
SEMA3A|GAL	SEMA3A	GAL	-0.6758144
SEMA3B|LTBP4	SEMA3B	LTBP4	0.06249894
SEMA3B|FGFRL1	SEMA3B	FGFRL1	0.44268731
SEMA5A|C5AR1	SEMA5A	C5AR1	0.26480471
EDNRA|STC2	EDNRA	STC2	-0.7028178
PLXNB1|FGFRL1	PLXNB1	FGFRL1	0.14262903
ANGPTL2|SORT1	ANGPTL2	SORT1	-0.1548456

### Verification of IRGP signature

In the GSE21257 data set, patients were divided into high-risk group and low-risk group according to the same signature and the same cut-off value. As shown in Figure 1C, patients in the low-risk group have a longer overall survival. After adjusting for age, gender and other variables, the results of multivariate Cox regression showed that IRGP signature is an independent prognostic factor (HR:1.560, 95%CI: 1.033-2.354, P= 0.034). As shown in Figure 3, IRGP signatures still have excellent prediction capabilities in the verification set (AUC=0.918).

### Correlation between IRGP and clinical characteristics

**Figure 4:**
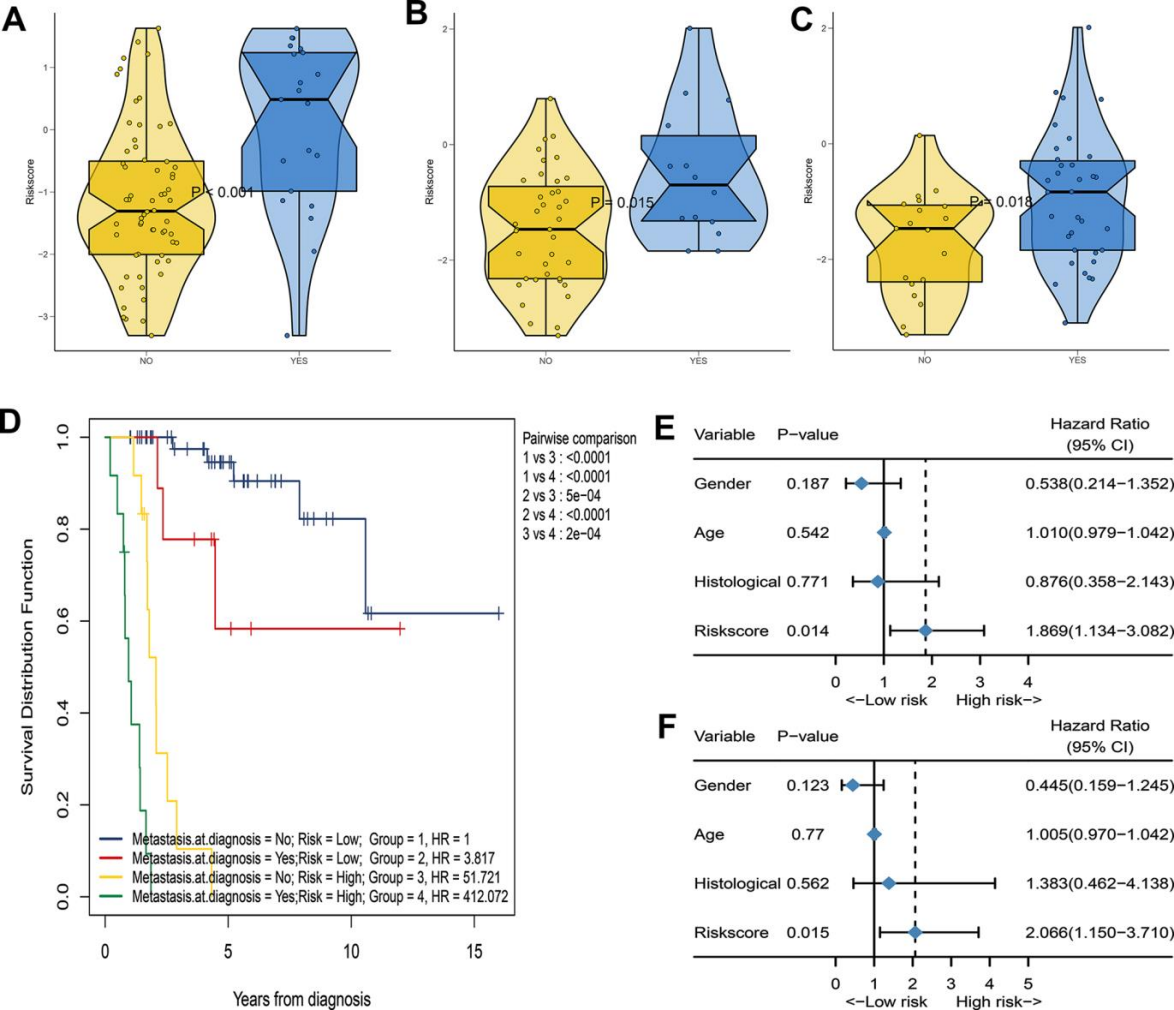
**Assess the correlation between IRGP signature and metastasis status.** (**A**) Box violin plot of the relationship between the metastasis status at diagnosis and the IRGP signature value in the TCGA cohort. (**B**) Box violin plot of the relationship between the metastasis status at diagnosis and the IRGP signature value in the GSE21257 cohort. (**C**) Box violin plot of the relationship between the metastasis status within 5 years and the IRGP signature value in the GSE21257 cohort. (**C**) Kaplan–Meier curves of overall survival for patients in TCGA cohort stratified by both IRGP signature, and metastasis status. (**D**) Forest plot of univariate Cox regression results between various variables and tumor metastasis in the GSE21257 cohort. (**E**) Forest plot of univariate Cox regression results between various variables and tumor metastasis in the GSE21257 cohort. (**F**) Forest plot of multivariate Cox regression results between various variables and tumor metastasis in the GSE21257 cohort.

We further analyzed the relationship between IRGP signatures and clinical characteristics. We divided patients into different subgroups based on clinical variables. As shown in Figure 2E, IRGP signatures can predict the overall survival of patients in different subgroups. As shown in Figure 4A, in the TCGA cohort, patients in the metastasis group had higher IRGP values. In addition, based on the patient’s metastatic status and IRGP signature, we divided the patients in the TCGA data set into four groups. As shown in Figure 4D, there was no significant difference in overall survival between metastatic patients and non-metastatic patients in the low-risk group. Among patients in the metastasis group, patients in the low-risk group had a longer overall survival than those in the high-risk group. The GSE21257 cohort simultaneously recorded the metastatic status of the patient at the time of the initial diagnosis and the status within 5 years after the diagnosis. Therefore, in the GSE21257 cohort, we investigated the relationship between IRGP signatures and these two tumor metastasis states. As shown in Figure 4B, 4C, both in the initial diagnosis and within 5 years of diagnosis, patients in the metastasis group had higher IRGP signature values. Finally, we excluded patients who had metastases at the time of diagnosis. Based on the time of sarcoma metastasis, the occurrence of sarcoma metastasis is defined as the outcome. As shown in Figure 4E–4F, patients with high IRGP signatures have a higher risk of metastasis. The results of multivariate analysis indicate that IRGP signature is an independent risk factor.

### Construction and evaluation of nomogram based on IRGP signature

**Figure 5:**
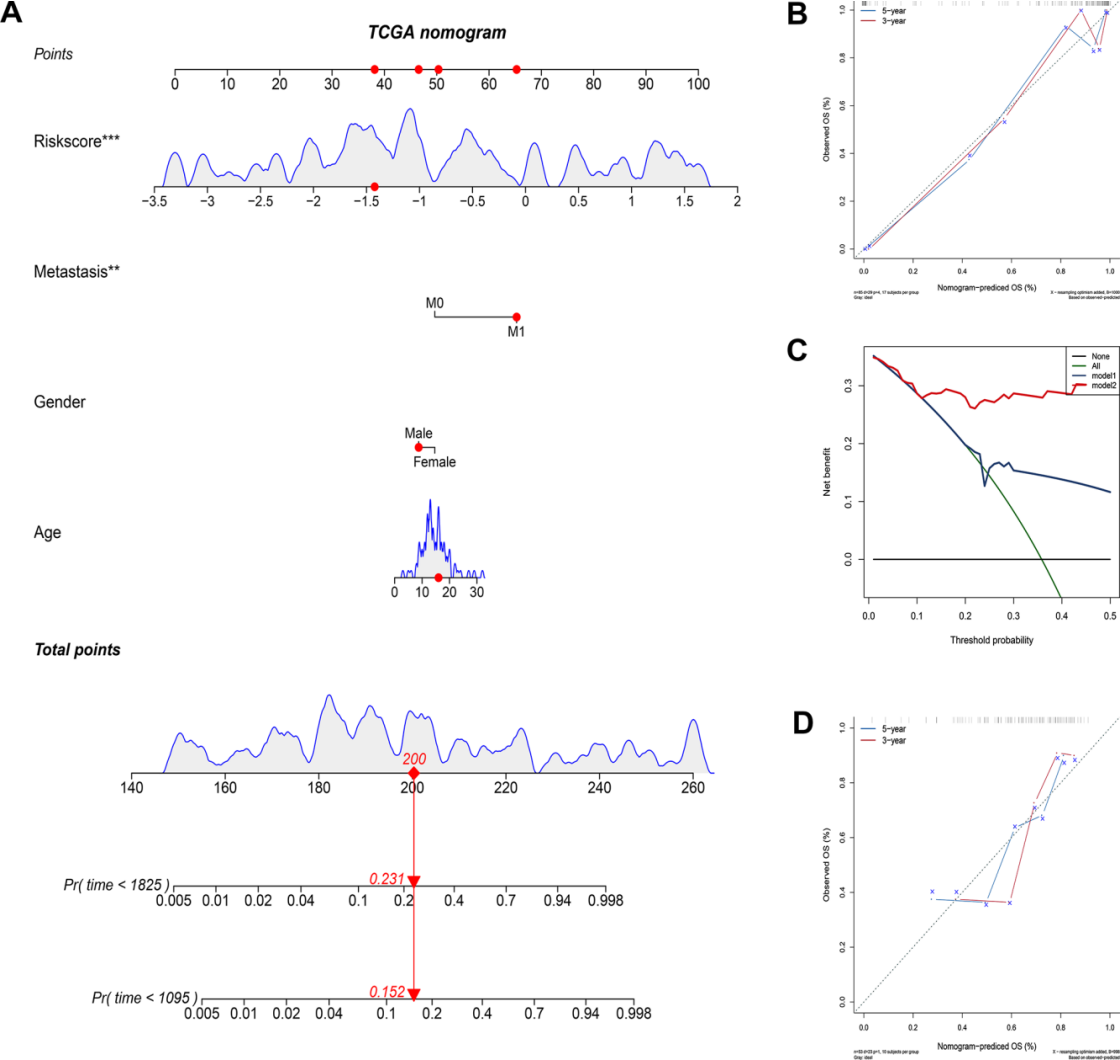
**Construct and evaluate nomograms in TCGA and GSE21257 cohort.** (**A**) Nomogram to predict the probability of TCGA patients mortality based on IRGP and clinical variables. (**B**) The calibration plot for internal validation of the nomogram. (**C**) Decision curve analyses of the nomograms based on IRGP signature for 3-year overall survival. (**D**) The calibration plot of the nomogram in the GSE21257 data set is used for external verification. *P<0.05, **P<0.01, ***P<0.001.

We constructed a nomogram based on the clinical variables and IRGP signatures of the TCGA dataset. Subsequently, the nomogram was further verified in the GSE21257 dataset. The results of the calibration chart show that the nomogram performance is the best in predicting the 3-year OS in the two cohorts. In the TCGA cohort, the nomogram C index was 0.903, while in the GSE21257 cohort, the nomogram C index was 0.702. The result of the calibration curve shows that the nomogram has good discrimination ability. The results of the decision curve analysis show that the combination model can bring net benefits in predicting overall survival (Figure 5).

### Relationship between IRGP signature and immune cell infiltration

**Figure 6:**
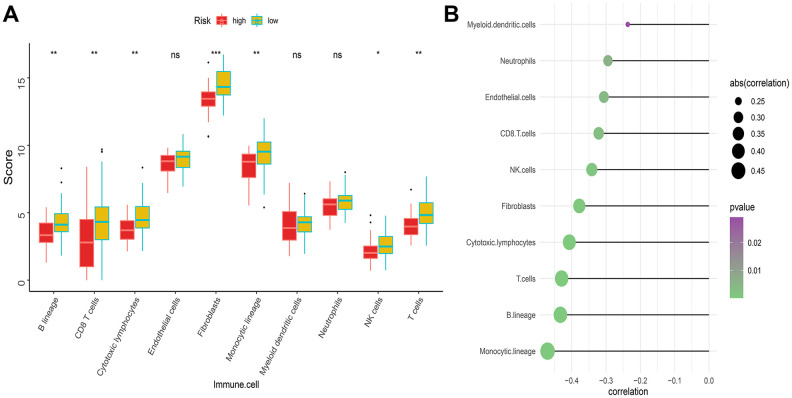
**Difference of immune infiltration among patients in IRGP signature group.** (**A**) Box plot showing the absolute abundance scores of 10 immune cell and stromal cell populations in two groups of patients. (**B**) Correlation matrix between absolute abundance scores of immune cells and stromal cells and IRGP values. The size of the bubble represents the degree of correlation and the color of the bubble represents the p-value of the correlation. (ns represents no significance, *P < 0.05, **P < 0.01, ***P < 0.001).

**Figure 7:**
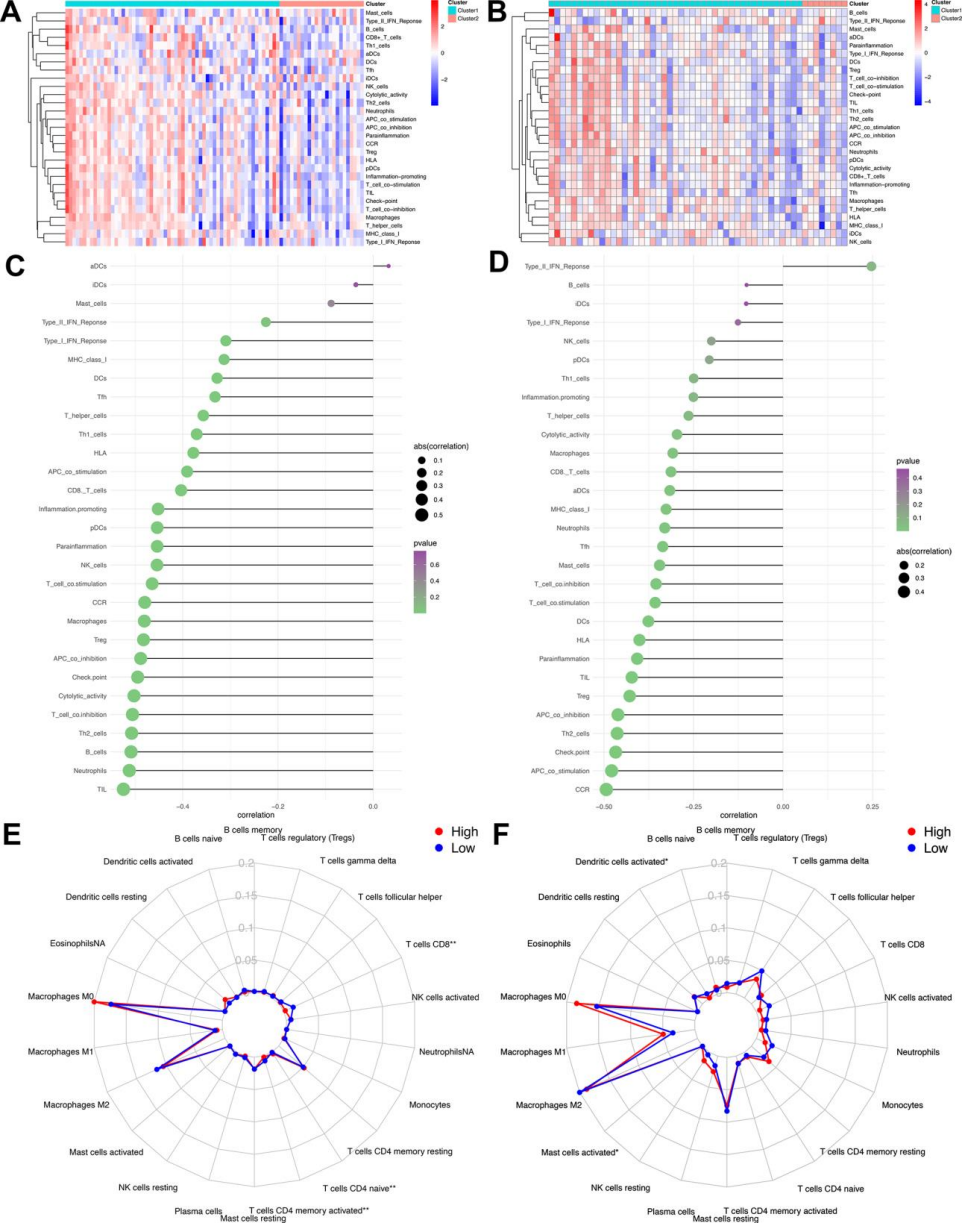
**Assess the relationship between immune microenvironment and IRGP signature.** (**A**) Heat map of results of single sample gene set enrichment analysis in TCGA cohort. (**B**) Heat map of results of single sample gene set enrichment analysis in GSE21257 cohort. (**C**) Relationship between 29 immune-related gene sets and IRGP signature values in TCGA cohort. (**D**) Relationship between 29 immune-related gene sets and IRGP signature values in GSE21257 cohort. (**E**) Radar chart of the relationship between 22 immune cell infiltration and IRGP signature grouping in TCGA cohort. (**F**) Radar chart of the relationship between 22 immune cell infiltration and IRGP signature grouping in GSE21257 cohort.

As mentioned above, the abundance of 10 immune-related cells was calculated using the MCP-counter method. As shown in Figure 6A, a significant difference was observed between the two groups of patients. Compared with patients in the high-risk group, the abundance of the 7 cell populations in the patients in the low-risk group was higher (B-cell lineage, CD8+T cells, Cytotoxic lymphocytes, Fibroblasts, Monocytic lineage cells, NK cells, T cells). In addition, as shown in Figure 6B, the degree of immune cell infiltration is negatively correlated with the IRGP value. Similarly, the results of ssGSEA showed that in the high-risk group, most of the 29 immune-related gene sets or immune cells scored lower. We further explored the relationship between these immune-related gene sets or immune cells and the IRGP signature value. As shown in Figure 7, most gene sets or immune cell scores are negatively correlated with IRGP signature values. Subsequently, we used CIBERSORT to infer the relative proportion of 22 infiltrating immune cells in each sample. As shown in Figure 7E, 7F, in the TCGA cohort, the relative proportion of T cells CD8 and T cells CD4 memory activated was higher in the low-risk group, while the relative proportion of T cells CD4 navie was higher in the high-risk group. In the GSE21257 cohort, only the relative abundance of Mast cells activated and Dendritic cells activated differed. Although the radar chart showed that the relative abundance of T cells CD8 was higher in the low-risk group, it did not reach a statistical difference. It is worth noting that the infiltration ratio of M0 macrophages and M2 macrophages is higher in all patients.

### Relationship between IRGP signature and immunotherapy efficiency

**Figure 8:**
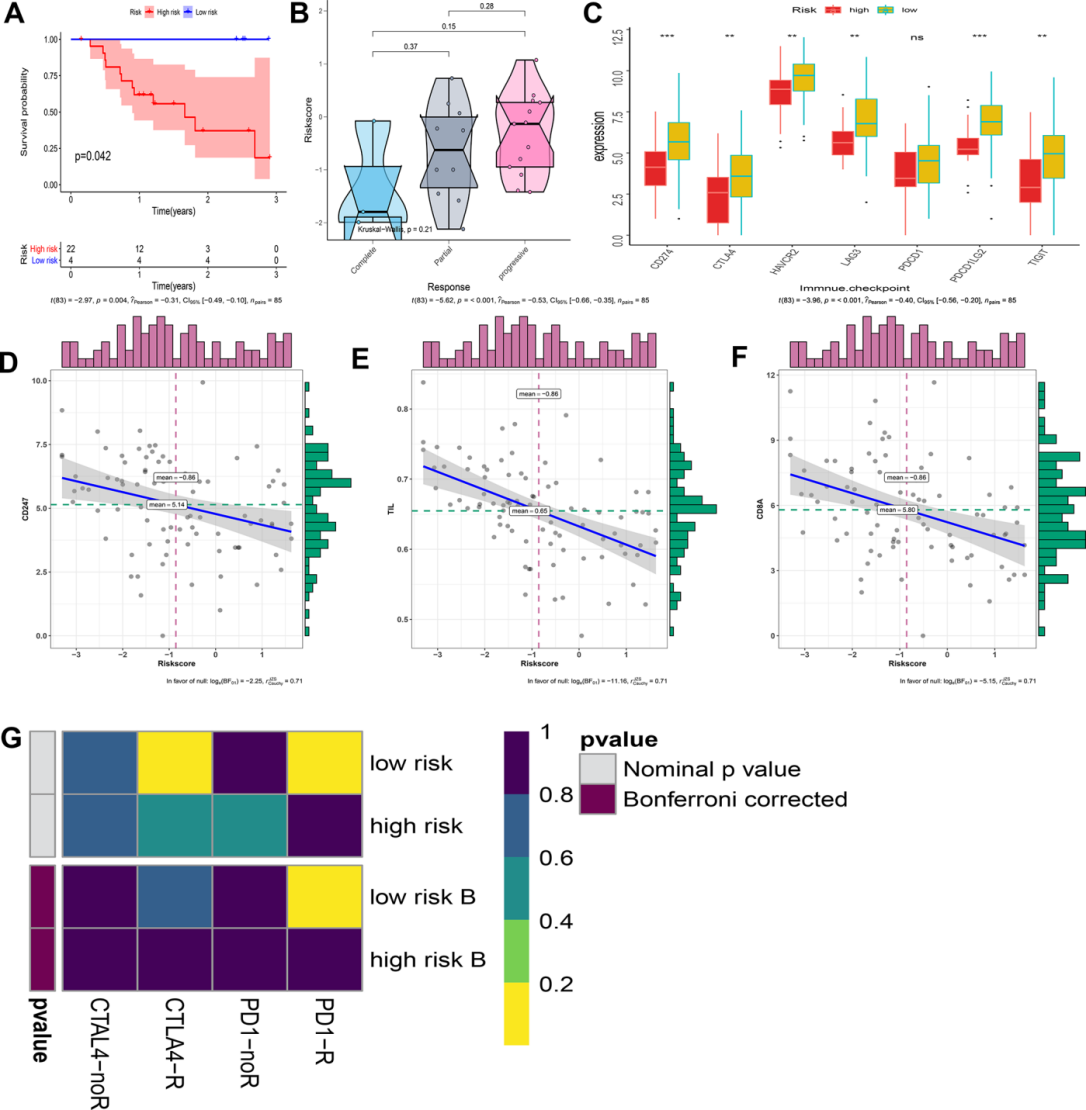
**Explore patients who may hope to benefit from immunotherapy.** (**A**) Kaplan–Meier curves of overall survival according to IRGP groups in GSE78220. (**B**) Box violin plot of the relationship between the immunotherapy response and the IRGP signature value in the GSE78220 cohort. (**C**) Box plot showing the expression of 7 immune checkpoint genes in two groups of patients. (**D**) Correlation between IRGP value and CD247 gene expression. (**E**) Correlation between IRGP value and TIL. (**F**) Correlation between IRGP value and CD8A gene expression. (**G**) Heatmap of correlation between expression profiles of patients in the IRGP group and patients receiving immunotherapy. The color of the grid represents the correlation P-value.

Considering that our signature is based on IRGP, we further explored the relationship between IRGP signature and immunotherapy response. First, we tested the predictive ability of IRGP signatures in the GSE78220 data set. We used IRGP signatures to divide patients into high-risk and low-risk groups. Kaplan-Meier curves show that patients in the high-risk group have a poorer prognosis than those in the lower-risk group. In addition, IRGP signature values of patients with poor immunotherapy response showed an increasing trend. Unfortunately, it did not reach statistical significance (Figure 8A, 8B).

Subsequently, we explored the expression of 7 immune checkpoint genes in two groups of patients. As shown in Figure 8C, except for the PDCD1 gene, all other immune checkpoint genes are highly expressed in the low-risk group. We further explored the relationship between IRGP signature and TMIT. Since there is no optimal cutoff value for TMIT classification, we regard the IRGP signature as a continuous variable. Our results show that the IRGP signature value is negatively correlated with TIL and the expression of CD8A gene and CD247 gene. Therefore, patients in the low-risk group are more likely to be classified as TMIT I.

Finally, we used SubMap analysis to further study the relationship between IRGP signature and immunotherapy efficiency. Using subclass mapping, the expression profiles of the two groups of patients (high-risk group and low-risk group) were compared with a published immunotherapy data set. This data set records the expression data of 47 melanoma patients treated with programmed cell death protein 1 (PD-1) immune checkpoint inhibitors or cytotoxic T lymphocyte associated protein 4 (CTLA-4) immune checkpoint inhibitors. The results showed that the expression profiles of patients in the low-risk group were correlated with those in the PD-L1 response group. This indicates that patients in the low-risk group are more likely to benefit from PD-L1 therapy (Figure 8G).

### Gene set enrichment analysis

**Figure 9:**
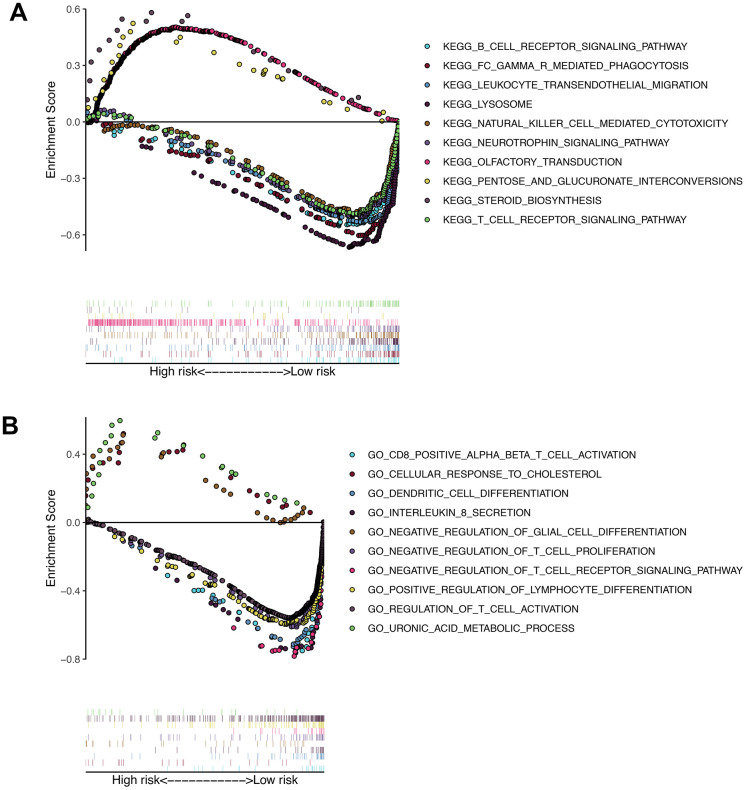
**Results of gene set enrichment analysis in the TCGA cohort.** (**A**) The significantly enriched KEGG pathways in TCGA cohort by GSEA. (**B**) The significantly enriched GO terms in TCGA cohort by GSEA.

Gene set enrichment analysis was performed in patients in the high-risk group and the low-risk group. As shown in Figure 9, a large number of immune-related gene sets are enriched in the low-risk group, while the gene sets enriched in the high-risk group are those that are not related to the immune process.

## DISCUSSION

The immune system has been shown to play a key role in the occurrence and development of tumors [19, 20]. Immunotherapy has also shown great potential in a variety of tumors [11, 21]. Therefore, it is of great significance to study the clinical value and potential molecular mechanism of immune-related genes in osteosarcoma. Although many signatures have recently been developed that can predict patient prognosis, these signatures still have some deficiencies in overcoming the batch effects of different platforms. In addition, most of these signatures require pretreatment of gene expression profiles, which affects the widespread use of signatures. In this study, we developed and verified an immune-related gene signature that can predict the overall survival of osteosarcoma patients. The signature of the immune gene pair is derived from the pairwise comparison of gene expression in the same sample, which can be used more widely across different detection platforms [16]. Our signature is composed of 14 immune-related gene pairs, and patients are divided into high-risk group and low-risk group according to the calculated cut-off value. Our results show that the overall survival of patients in the high-risk group is significantly reduced. In addition, the results of univariate and multivariate Cox proportional hazards regression analysis indicate that signature is an independent prognostic factor in predicting the overall survival of osteosarcoma patients. In addition, we found that combining the signature and clinical characteristics to construct a nomogram can more accurately predict the patient’s overall survival and bring net benefits. Finally, we divided the patients in the verification group into a high-risk group and a low-risk group based on the same cutoff value and obtained the same results.

Today, clinical characteristics such as metastatic status are still the most important basis for doctors to stratify risk and make clinical decisions for patients with osteosarcoma [22]. Therefore, we further explored the relationship between signatures and clinical characteristics. Our results show that whether in the TCGA cohort or the GSE21257 cohort, the IRGP signature value of patients in the metastatic group is higher than that in the non-metastatic group. In addition, we divided the patients into 4 groups according to the metastasis status and IRGP signature, and then stratified the patients more accurately. Our results indicate that among the patients in the IRGP signature low-risk group, there was no significant difference in overall survival between patients in the metastatic group and those in the non-metastatic group. Among patients in the metastasis group, patients in the low-risk group had better overall survival than those in the high-risk group. This also explains why patients with similar clinical characteristics show completely different clinical outcomes despite undergoing the same treatment. Therefore, combining the IRGP signature with traditional clinical features can more accurately stratify patients and formulate individualized treatment plans. In addition, identifying patients with a high risk of metastasis and enhancing follow-up of these patients may improve the prognosis of osteosarcoma patients. We further explored the relationship between IRGP signature and tumor metastasis in the GS21257 cohort. Our results indicate that IRGP signature is an independent risk factor for tumor metastasis. Patients with high IRGP signature values have an increased risk of tumor metastasis within five years of diagnosis. Therefore, it is necessary to strengthen the follow-up of patients with high IRGP signature value. It is worth noting that due to the limitation of the sample size, further research is needed to prove our conclusion.

Recently, immune checkpoint inhibitor therapy has shown promising clinical benefits [23, 24]. Historically, sarcomas were the first tumor model for which immunotherapy was suggested as a relevant therapeutic strategy [25]. Unfortunately, recent studies have shown that PD-1 inhibitors have limited activity in osteosarcoma. It is important to identify patients who may benefit from this treatment strategy. [9, 11, 26] Therefore, we used three methods to evaluate the relationship between IRGP signature and immunotherapy efficiency. First, we found that IRGP signatures can predict the outcome of patients with metastatic melanoma who received anti-PD-L1 treatment and that patients with poor treatment response showed an increasing trend in IRGP signature values. However, due to sample size limitations, this result must be interpreted with caution. Subsequently, we further explored the relationship between IRGP signature and TMIT. Similarly, patients with low IRGP signature values are more likely to be classified as TIMT type I due to high PD-L1 gene expression and high CD8A gene expression, and therefore are more likely to benefit from anti-PD-1 therapy. Interestingly, a recent study showed that the expression of PD-L1 gene is related to the poor prognosis of osteosarcoma patients, which is contrary to our conclusion [27]. Therefore, we used univariate Cox regression to explore the relationship between PD-L1 gene expression and the overall survival of osteosarcoma patients. The results show that PD-L1 gene expression is a protective factor for the overall survival of osteosarcoma patients (Supplementary Figure 1). Therefore, further research is needed to verify the relationship between PD-L1 gene expression and the prognosis of osteosarcoma patients. Finally, we compared the expression profiles of TCGA patients with melanoma patients treated with immune checkpoint inhibitors by submap analysis. There is a certain correlation between the expression profiles of the low-risk group and the PD-L1 response group. In summary, we speculate that patients in the low-risk group may benefit from anti-PD-L1 therapy. However, it is necessary to further study the relationship between IRGP signature and immunotherapy efficiency in osteosarcoma patients.

Our results show differences in biological processes and immune infiltration between the two groups. Among them, the results of GSEA show that a large number of immune-related pathways are enriched in the low-risk group, while immune-unrelated pathways are enriched in the high-risk group. From this point of view, patients in the low-risk group are also more likely to benefit from immunotherapy. The results of MCP-counter and CIBERSORT showed that there was also a difference in immune cell infiltration between the two groups. Among them, CD8T cells were more infiltrated in the low-risk group. Consistent with our conclusion, CD8 + T lymphocyte infiltration has a positive effect on the prognosis of various tumors, such as hepatocellular carcinoma and breast cancer [28, 29]. In addition, CD8 + T lymphocyte infiltration has been found to improve the prognosis of patients with osteosarcoma [30]. At the same time, a high proportion of M0 and M2 macrophage infiltration existed in both groups. Interestingly, despite many recent studies on tumor-associated macrophages, the conclusions do not seem to be consistent. Many studies have shown that macrophages are usually associated with immunosuppressive microenvironments. In addition, a high number of M2 macrophages have been shown to be associated with poor prognosis in various tumors [31–33]. A recent study also showed that macrophages reduce the sensitivity of osteosarcoma to neoadjuvant chemotherapy drugs by secreting interleukin1β [34]. Interestingly, the results of Buddingh et al. indicate that in the case of osteosarcoma, the direct or indirect antitumor activity of macrophages exceeds its possible tumor supporting effect [35]. In addition, the results of Anne Gomez-Brouchet et al. showed that, contrary to the results in other solid tumors, the presence of CD163-positive M2-polarized macrophages is essential for inhibiting osteosarcoma progression [36]. Therefore, it is necessary to further study the role of macrophages in osteosarcoma.

Our signature consists of 21 immune-related genes, and previous studies have described the value of some genes in osteosarcoma. Both ANGPTL2 and ANGPTL4 belong to the Angiopoietin-like protein family. Previous studies have shown that ANGPTL2, as a chronic inflammatory mediator, is overexpressed in various tumors and is associated with poor prognosis [37, 38]. In addition, studies have shown that ANGPTL2 is highly expressed in osteosarcoma cells induced by hypoxia / HIF-1α and promotes cell proliferation, invasion, migration and G1 phase arrest. ANGPTL2 also enhances the expression of VEGFA, Ang II and HK2 in mice to enhance angiogenesis and glycolysis [39]. Similarly, ANGPTL4 is highly expressed in hypoxic-induced osteosarcoma cells and promotes osteosarcoma cell proliferation and migration as well as osteoclast formation and bone resorption activity [40]. Previous studies have shown that the CCL2 gene affects the proliferation of osteosarcoma cells through the RANKL signaling pathway. In addition, the expression of CCL2 gene in high-grade osteosarcoma cells increased and promoted the proliferation and invasion of osteosarcoma cells [41]. SEMA3A can inhibit the ability of osteosarcoma cells to stimulate osteoclast production [42]. The signatures based on these gene pairs respond well to the patient’s immune status.

It should be admitted that our research still has some limitations. First, although the incidence of osteosarcoma is relatively low, the study involved a small sample size. Second, the conclusion about the efficacy of immunotherapy cannot be verified in osteosarcoma patients. Further research is needed to verify our results. Finally, this study is a retrospective study, and further prospective studies are needed to verify our results.

In conclusion, IRGP signatures can accurately predict the overall survival of osteosarcoma patients and combining signatures with clinical characteristics can bring net benefits. In addition, signatures may identify patients who are more likely to benefit from immunotherapy. Further research is needed to verify our conclusion.

## MATERIALS AND METHODS

### Clinical samples and data acquisition

We downloaded the level three RNA-Seq expression data of 88 osteosarcoma patients from TCGA database (https://portal.gdc.cancer.gov) (FPKM). Subsequently, the latest clinical data of osteosarcoma patients were downloaded from the TARGET database (https://ocg.cancer.gov/programs/target). Finally, 85 patients with both RNA-seq expression data and valid clinical information were identified for analysis in this study. We retrieved the osteosarcoma dataset with clinical information such as overall survival on the Gene Expression Comprehensive Library (GEO; http://www.ncbi.nlm.nih.gov/geo/). We then downloaded the GSE21257 dataset on the GPL10295 platform (Illumina human-6 v2.0 expression beadchip) [35]. This data set is the only one in the osteosarcoma data set that has the overall survival of the patient and was used to verify our results. Finally, for the analysis of immunotherapy efficiency, the GSE78220 data set (GPL11154 platform (Illumina HiSeq 2000 (Homo sapiens)) was downloaded. GSE78220 dataset records transcriptome data of 28 patients with metastatic melanoma treated with anti-PD-1 agents (pembrolizumab or nivolumab). Supplementary Table 5 provides detailed clinical information for the GSE78220 data set. If a target gene corresponds to multiple probes, the average expression value of the probes is used to represent the expression level of the gene. At the same time, delete genes whose expression is 0 in all samples.

### Identification of prognostic-related IRGPs in patients with osteosarcoma

We downloaded the Immunity Related Gene List (IRG) from the Immunology Database and the Analysis Portal (ImmPort) database [43]. The list contained a total of 1811 unique IRGs, which are related to T cell receptor and B cell antigen receptor signaling pathways, cytotoxicity of natural killer cells, antigen processing and presentation pathways. First, immune-related genes with high variability were identified. Specifically, a particular gene is considered to have high variability if it has a high median absolute deviation (MAD>0.5) value in each dataset. We then used the gene expression levels of these genes in each sample for pairwise comparison to construct IRGP. In a specific sample, if the expression value of the first IRG is greater than that of the second IRG, the score of this IRGPs in the sample is 1, otherwise it is 0. The score of each IRGP in all samples was calculated and the IRGPs with low variation were removed (IRGP with a score of 1 or 0 in more than 80% of the sample in any data set). Finally, IRGPs with higher variability were identified for further analysis. Use TCGA cohort as training set. Univariate Cox regression analysis was performed on these IRGPs in the TCGA cohort, and IRGPs with p <0.005 were considered as prognostic-related IRGPs and used for subsequent analysis.

### Construction and evaluation of signatures based on IRGPs

Lasso Cox proportional hazards regression analysis was performed on the above-mentioned prognostic-related IRGPs, and finally an optimal model composed of 14 gene pairs was determined. Subsequently, the optimal-model based IRGP signature of each patient was calculated. In the 3-year overall survival TCGA cohort, time-dependent receptor operating characteristic (ROC) curve analysis was used to determine the optimal cut-off value for IRGP signature [44]. According to the cut-off value of IRGP signature, patients were divided into high-risk group and low-risk group. The log-rank test was used to evaluate the overall survival difference between the low-risk group and the high-risk group and the KM survival curve was drawn. Receiver operating characteristic (ROC) curve analysis was used to evaluate the sensitivity and specificity of IRGPs. A ROC curve including clinical characteristics was drawn, and the area under the curve (AUC) was calculated. Finally, univariate and multivariate Cox regression analysis was used to investigate whether the prognostic value of IRGP was affected by other clinical characteristics.

### Verification of signatures based on IRGPs

In order to verify the IRGPs signatures, we calculated the risk score of each patient in the GSE21257 data set using the above method and divided the patients into a high-risk group and a low-risk group according to the cutoff value above. The log-rank test was then used to assess the overall survival difference between the two groups and to plot the KM survival curve. We used univariate and multivariate Cox regression to evaluate the independent prognostic value of signatures. Finally, the receiver operating characteristic (ROC) curve was drawn and the area under the curve (AUC) was calculated.

### Evaluation of the relationship between signatures and clinical characteristic

We evaluated the relationship between IRGPs signature and clinical characteristics. First, we evaluated the relationship between the IRGP signature value and clinical variables. Subsequently, we divided patients with osteosarcoma into different subgroups based on clinical variables and explored the prognostic value of IRGP signatures among different subgroups. In addition, we divided patients in the TARGT-OS cohort into four groups based on their metastatic status and IRGP group. The differences in overall survival between the four groups of patients were evaluated. Finally, the relationship between IRGP signature and tumor metastasis was further evaluated in the GSE21257 cohort.

### Construction and evaluation of nomograms

We combined the clinical characteristics of the TCGA data set with the IRGP signature to construct a nomogram and verified the nomogram using the GSE21257 data set as external verification We used the C index to evaluate the discriminative power of the nomogram and drew a calibration chart to evaluate the accuracy of the nomogram. We then compared the decision curve analysis between the clinical characteristics model and the combined model including gene signature and clinical characteristics.

### Estimation of immune infiltration

First, the immune infiltration assessment was performed using the “microenvironment cell population count (MCP-counter)” method [45]. Using the normalized FPKM expression matrix converted by log2 as input, the absolute abundance scores of ten immune cell and stromal cell populations are generated through the “MCP-counter” package. Research shows that immune cell infiltration assessed by MCP-counter algorithm performs well when comparing between samples [46]. Subsequently, CIBERSORT was used to infer the relative proportion of 22 infiltrating immune cells in each sample for supplementation. In addition, the single sample GSEA was used to evaluate the enrichment of 29 immune-related gene sets in each sample.

### Relationship between IRGP and immunotherapy efficiency

First, verify the IRGP signature in the GSE78220 cohort. Considering the significant heterogeneity between different tumors, we recalculated the cut-off value using time-dependent ROC curve analysis. According to the cut-off value, divide the patients into high-risk group and low-risk group and draw KM survival curve. Subsequently, ‘Tumor microenvironment immune type (TMIT)’ was used to speculate the efficacy of anti-PD-1 / PD-L1 treatment [47, 48]. TMIT divided patients into four types based on PD-L1 and CD8A mRNA expression, which has been shown to predict patients’ response to immune checkpoint inhibitors in pan-cancer analysis. At the same time, we also explored the differences in gene expression of the other six immune checkpoints between the two groups of patients. In addition, we used SubMap analysis (Gene Pattern) to compare gene expression profiles of osteosarcoma patients with melanoma patients treated with immunotherapy to indirectly predict the efficacy of immunotherapy in osteosarcoma patients [49, 50].

### Gene set enrichment analyses

GSEA software (version 4.0.1) was used to perform gene set enrichment analysis between high-risk and low-risk groups. Recognized the enriched terms in gene ontology (GO) and KEGG in high-risk group and low-risk group respectively. P <0.05 and False discovery rate (FDR) <0.05 are considered statistically significant.

### Statistical analysis

Except for gene set enrichment analysis, all statistical analyses involved in this research were conducted through R software (version 3.6.3, R Foundation for Statistical Computing, Vienna, Austria). If no special instructions, p <.05 is considered statistically significant.

### Data availability statement

RNA-seq data of the TCGA cohort can be obtained from the TCGA database (https://portal.gdc.cancer.gov). Clinical data of these patients can be obtained from the TARGET database (https://ocg.cancer.gov/programs/target). The data of GSE21257 can be obtained from the GEO database (https://www.ncbi.nlm.nih.gov/geo/query/acc.cgi?acc=GSE21257).

## Supplementary Material

Supplementary Figure

Supplementary Table 1

Supplementary Table 2

Supplementary Table 3

Supplementary Tables 4 and 5
