# Supplementary material for: Construction of immune-related gene pairs signature to predict the overall survival of osteosarcoma patients
Source: Aging (Albany NY). 2020 Nov 16;12(22):22906–26. doi: 10.18632/aging.104017 (PMC7746392; doi:10.18632/aging.104017)
Supplement: Supplementary Figure [file aging-12-104017-s001..pdf]

SUPPLEMENTARY FIGURE

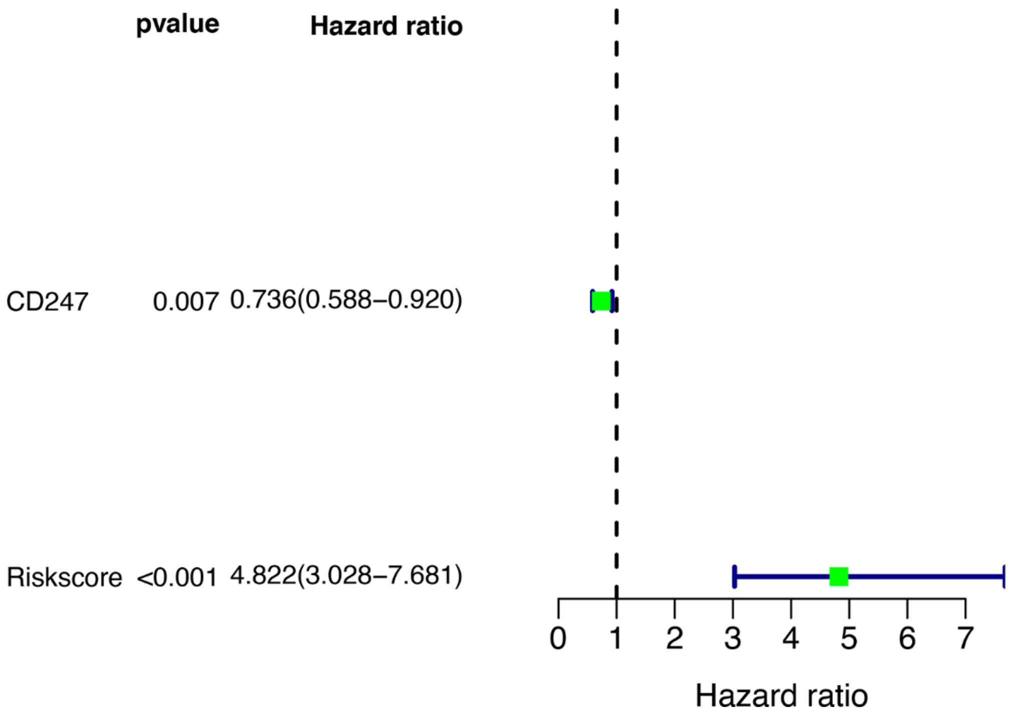

Supplementary Figure 1. Results of univariate COX regression analysis of PD-L1 and Riskscore.
