# Supplementary material for: Construction of immune-related gene pairs signature to predict the overall survival of osteosarcoma patients
Source: Aging (Albany NY). 2020 Nov 16;12(22):22906–26. doi: 10.18632/aging.104017 (PMC7746392; doi:10.18632/aging.104017)
Supplement: Supplementary Tables 4 and 5 [file aging-12-104017-s005..pdf]

SUPPLEMENTARY TABLES

Supplementary Table 4. Results of Univariate Cox regression of IRGP with higher variation. (P<0.005).

IRGP	HR	HR.95L	HR.95H	coxPvalue
FCER1G|NDRG1	0.27639592	0.12554102	0.60852383	0.00140533
HLA-DQB1|STC2	0.23444675	0.09967148	0.55146446	0.0008881
APOBEC3G|GAL	0.33497266	0.1591734	0.70493365	0.0039641
FABP4|GAL	0.23429033	0.09975861	0.55024781	0.00086442
OASL|GAL	0.33794111	0.1617213	0.70617906	0.00391252
APOD|CCL2	3.03038955	1.43686979	6.39115728	0.00359138
TFRC|ANGPTL2	3.63343365	1.69767116	7.77644128	0.00088986
TFRC|TNFRSF21	3.00990706	1.42652227	6.35078797	0.00382254
F2R|SEMA3B	0.32349419	0.15533762	0.67368412	0.00256725
F2R|ANGPTL4	0.29288792	0.13270808	0.64640626	0.00236392
CCL5|SEMA3B	0.2096095	0.07978568	0.55067705	0.0015214
WNT5A|GAL	0.28471691	0.13684325	0.59238377	0.00077754
HMOX1|FOS	0.32346229	0.15451936	0.67711811	0.00274979
HCK|SEMA5A	0.28401534	0.12572024	0.64162071	0.00246845
HCK|PLXNB1	0.27935222	0.12343848	0.63219887	0.00221056
HCK|GAL	0.28460063	0.13388452	0.60498048	0.00109025
HCK|STC1	0.20276464	0.07029161	0.58489914	0.00315527
RAC3|FGFRL1	7.15301218	2.12340384	24.0960209	0.00149745
SEMA3A|SEMA4D	0.33542642	0.16001111	0.70314419	0.00382089
SEMA3A|GAL	0.22876178	0.10756573	0.48651139	0.00012742
SEMA3A|TNFRSF11B	0.31504209	0.1457478	0.68098126	0.003315
SEMA3B|LTBP4	3.63638195	1.74042023	7.59774764	0.00059502
SEMA3B|VEGFC	2.95306523	1.3886214	6.28003733	0.00491149
SEMA3B|C3AR1	3.7431678	1.51781114	9.2312573	0.00415712
SEMA3B|FGFRL1	4.25029395	1.96251825	9.20500926	0.00024253
SEMA5A|C5AR1	3.93004313	1.59685975	9.67225772	0.00289627
C5AR1|PLXNB1	0.26887173	0.10918511	0.66210498	0.00428033
EDNRA|STC2	0.22184761	0.10620662	0.46340204	6.16E-05
PLXNB1|VEGFC	2.88263163	1.38280123	6.009226	0.00473213
PLXNB1|FGFRL1	3.23045085	1.51805887	6.87444531	0.00233969
IGF2|PDGFD	0.33598457	0.1612484	0.7000729	0.00359188
ANGPTL2|SORT1	0.2760354	0.13008073	0.5857558	0.00079861

Supplementary Table 5. Clinical information of patients in the GSE78220 data set.

ID	Gender	Age	OS	Status	Response
GSM2069823	F	66	607	DEAD	progressive
GSM2069824	M	55	927	ALIVE	Partial
GSM2069825	M	62	948	ALIVE	Partial
GSM2069826	M	61	439	ALIVE	Partial
GSM2069827	M	51	882	ALIVE	Partial
GSM2069828	F	55	662	DEAD	progressive
GSM2069829	M	69	NA	NA	Complete
GSM2069830	M	68	1054	ALIVE	Complete
GSM2069831	M	60	387	ALIVE	progressive
GSM2069832	M	59	327	DEAD	progressive
GSM2069833	F	53	917	ALIVE	Complete
GSM2069834	F	27	54	ALIVE	progressive
GSM2069835	M	70	980	DEAD	Partial
GSM2069836	M	19	186	DEAD	progressive
GSM2069837	M	45	1060	ALIVE	Partial
GSM2069838	F	63	337	DEAD	progressive
GSM2069839	M	55	182	DEAD	progressive
GSM2069840	M	63	103	DEAD	progressive
GSM2069841	M	74	262	DEAD	progressive
GSM2069842	M	83	548	ALIVE	Complete
GSM2069843	M	83	548	ALIVE	Complete
GSM2069844	M	82	439	DEAD	Partial
GSM2069845	M	84	269	DEAD	progressive
GSM2069846	M	47	704	ALIVE	progressive
GSM2069847	M	47	171	DEAD	progressive
GSM2069848	F	65	427	ALIVE	Partial
GSM2069849	F	70	364	ALIVE	Partial
GSM2069850	F	57	448	ALIVE	Partial
